# Centre-level fluid management practices in the BISTRO trial and their lack of association with participant fluid status and blood pressure in non-anuric haemodialysis patients

**DOI:** 10.1186/s12882-024-03837-y

**Published:** 2024-11-06

**Authors:** Neena Johal, Radha Sharma, John Belcher, David Coyle, Elizabeth J. Lindley, David Keane, Fergus J. Caskey, Indranil Dasgupta, Andrew Davenport, Ken Farrington, Sandip Mitra, Paula Ormandy, Martin Wilkie, Jamie Macdonald, Ivonne Solis-Trapala, Julius Sim, Simon J. Davies

**Affiliations:** 1 School of Medicine, Faculty of Medicine and Health Sciences, David Weatherall Building, Keele University, Keele, Staffordshire ST5 5BG UK; 2 NIHR Devices for Dignity, Sheffield Teaching Hospitals NHS Foundation Trust, Sheffield, UK; 3 Renal Medicine, Leeds Teaching Hospitals NHS Trust, Leeds, UK; 4 CÚRAM SFI Research Centre for Medical Devices, University of Galway, Galway, Ireland; 5 Population Health Sciences, University of Bristol, Bristol, UK; 6 Renal Medicine, University Hospitals Birmingham NHS Foundation Trust, Birmingham, UK; 7 UCL Department of Renal Medicine, Royal Free Hampstead NHS Trust, University College, London, UK; 8 Renal Medicine, East & North Hertfordshire NHS Trust, Hertfordshire, UK; 9 Manchester Academic Health Sciences Centre (MAHSC), University Hospital Manchester, Manchester, UK; 10 School of Health and Society, University of Salford, Manchester, UK; 11 Renal Medicine, Sheffield Teaching Hospitals NHS Foundation Trust, Sheffield, UK; 12 Institute of Applied Human Physiology, Bangor University, Bangor, North Wales UK

**Keywords:** Blood pressure, Bioimpedance, Fluid management, Hemodialysis, Comorbidity, Practice patterns

## Abstract

**Introduction:**

Fluid assessment and management is a key aspect of good dialysis care and is affected by patient-level characteristics and potentially centre-level practices. In this secondary analysis of the BISTRO trial we wished to establish whether centre-level practices with the potential to affect fluid status were stable over the course of the trial and explore if they had any residual associations with participant’s fluid status.

**Methods:**

Two surveys (S) of fluid management practices were conducted in 32 participating centres during the trial, (S1: 2017–18 and S2: 2021–22). Domains interrogated included: dialysate sodium concentration, (D-[Na^+^]), fluid and salt intake, residual kidney function, use of diuretics, incremental start, approaches to fluid assessment, management and dialysate temperature, (D-^o^C). Associations of these practices with the closeness of the participant’s post-dialysis target weight to their normally hydrated weight, pre- and post-dialysis systolic (SBP) and diastolic blood pressure, (DBP), were analysed using intra-class correlations and multilevel modelling with adjustment for visit, age, sex and comorbidity burden.

**Results:**

Variations in centre practices were reported but did not change during the trial, apart from some relaxation in salt and fluid restriction in S2. For our measures of fluid status, measured 2501 times in 439 non-anuric incident haemodialysis patients, centre-level intraclass correlations were extremely low, whereas patient-level correlations ranged between 0.12 and 0.47, strongest for pre- and post-dialysis-SBP, less so for post-dialysis-DBP. Multi-level analysis found no associations between D-[Na^+^], or assessment methods of fluid status. In S2, one centre, routinely using a D-C^o^ of 35°C had more divergence between the target and normally hydrated weight, but this was not observed in S1, and no other associations were found.

**Conclusions:**

Centre-level fluid management practices were stable over the course of the BISTRO trial, and in contrast to patient-level factors, no centre-level associations were detected with fluid status or blood pressure. This may be because the trial imposed a standardised approach to fluid assessment in all trial participants who at least initially had residual kidney function, potentially over-riding the effects of other centre practices. Survey responses revealed substantial scope for developing and evaluating standardised protocols to optimise fluid management.

**Supplementary Information:**

The online version contains supplementary material available at 10.1186/s12882-024-03837-y.

## Introduction

Fluid assessment and management is a key component of haemodialysis care [1, 2], and can be characterised as a complex intervention in which both individual patient-level clinical decisions are made, and centre-level practices and policies play a part [2–4]. The BISTRO trial, *BioImpedance Spectroscopy to maintain Renal Output*, was undertaken to establish whether bioimpedance added value to patient-level clinical decision making in the context of setting a post-dialysis target weight that avoids volume depletion where possible, so as to preserve residual kidney function for as long as possible [5]. Overall, knowledge of the bioimpedance data did not improve on clinical judgement in setting the post-dialysis target weight and inclusion of this information had no impact on the observed, albeit slower than anticipated, rate of loss in residual kidney function [6]. However, given that the trial was expected to run over several years it was considered important to assess if there were changes in centre-level practices related to fluid management over the course of the study that might affect its interpretation. Centre-level practices that have previously been considered important in managing fluid status include dialysate sodium concentration, dialysate temperature, use of blood volume monitoring and the use of standardised dialysis centre protocols, all of which have inconclusive evidence [2–4].

With this in mind, a survey of centre-level practices was developed and conducted twice during the trial. Here we describe the results of this survey and also look for any associations with two indicators of fluid status, the pre- and post-dialysis systolic and diastolic blood pressure and closeness of the trial participants’ target weight to their normally hydrated weight, in particular in association with the centre defaults for dialysate sodium concentration (D-[Na^+^]) and dialysate temperature, (D-^o^C), the routine use of fluid management protocols and routinely used methods for assessing fluid status.

## Methods

### Designing the survey content and administration

The survey was constructed by the BISTRO trial co-investigators prior to initiating recruitment, with expertise in fluid management, including input from expert patients – see supplementary materials for a full survey description as it was presented to the investigators. It was structured according to a number of domains that included: dialysate sodium concentration, diet and salt intake, residual kidney function, use of diuretics and incremental start, approaches to fluid assessment, approaches to fluid management, and dialysate temperature.

The survey was administered twice during the course of the trial, during the first year (S1: 2017–18) and last year (S2: 2020–21), so as to detect any significant changes in practices that might influence the trial outcomes. The surveys were nearly identical, with a single additional question on the use of isolated ultrafiltration only being asked in S2.

### Measurement of outcomes: fluid status and blood pressure

The trial design (ISCCTN number: 11342007) and primary outcomes of interest have been published [5, 6]. Briefly, throughout the trial, all participants had a pre-defined schedule of fluid assessments, which were made monthly, at 0, 1, 2 and 3 months and then every 3 months for up to two years, using a standardised proforma as defined in the trial protocol [5]. A few days prior to these clinical assessments all patients underwent an independent bioimpedance measurement (for details on the training see supplemental materials published with the trial outcomes) [6], using the Fresenius BCM device. From this data the bio-impedance estimated normally hydrated weight (BI-NHW) was recorded and presented to the clinician setting the clinical post-dialysis target weight (TW) in the intervention arm. On the day of the fluid assessment the routine measurement of the pre- and post-dialysis blood pressure was recorded. Summary data for these blood pressure readings, which were not different between the trial groups, were published in the main trial report [6].

### Statistical analysis

**Fig. 1:**
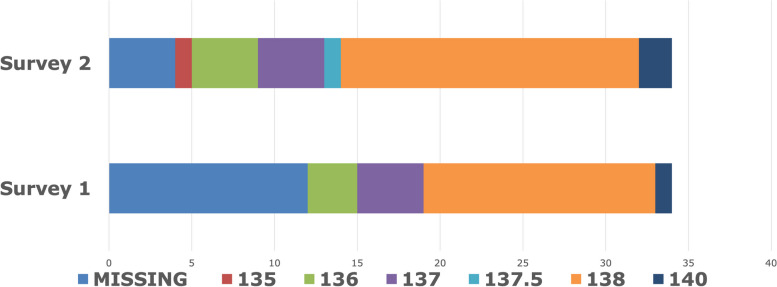
Distribution of default dialysate sodium concentration in the BISTRO participating sites

**Fig. 2:**
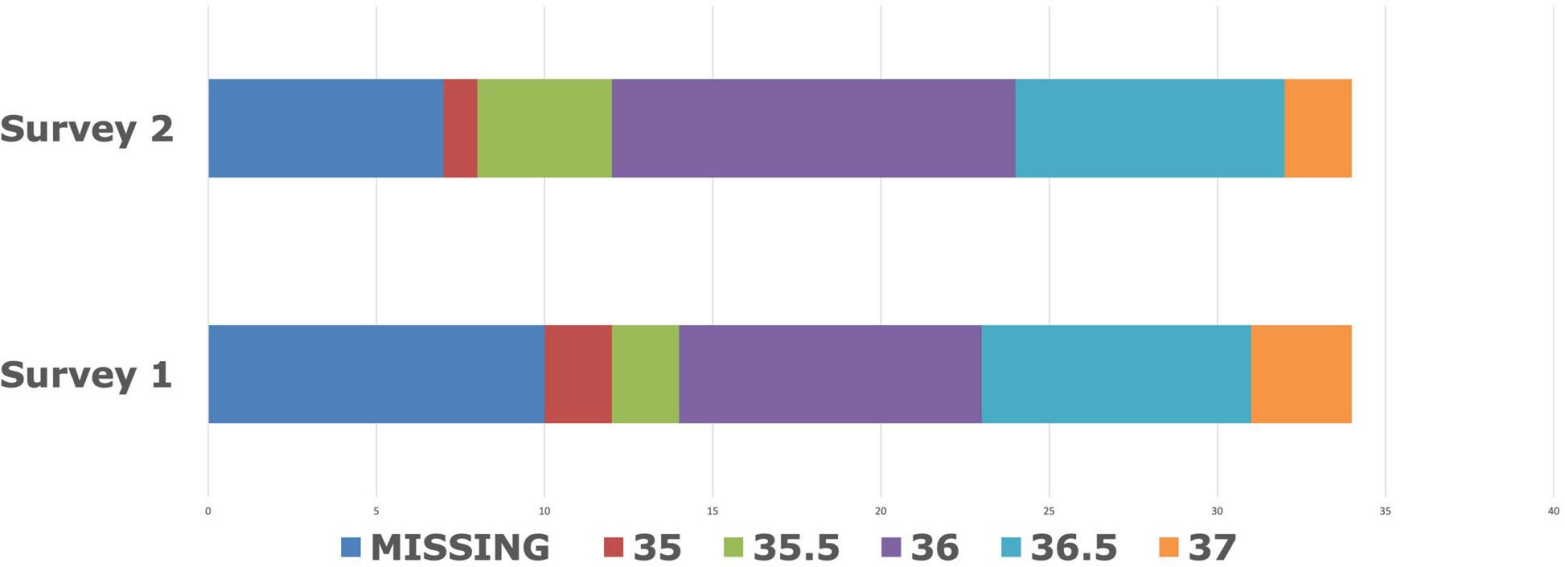
Distribution of default dialysate temperatures used in the sites participating in the BISTRO trial

Analysis of practice patterns in the BISTRO trial was planned prospectively. The findings of the two surveys are presented side-by-side for comparison using descriptive statistics appropriate to the question, but without between group statistical tests for difference as this would have involved multiple tests, in line with STROBE guidelines. The distribution of the default dialysate sodium concentrations and temperatures used by the centres is shown graphically, see Figs. 1 and 2. The surveys were not combined but analysed individually to establish whether there was consistency in any findings, and then compared to determine the level of agreement by question by calculating the percentage of responses that were the same. In some cases this required categorisation of the responses as appropriate (e.g. to the question ‘what proportion of patients have individualisation of their D-[Na^+^], there were two groups, ≤ 10% and > 10%). Patient outcome data (fluid status and blood pressure) were pooled from the two limbs of the trial, given the lack of between-group difference in the primary and secondary trial outcomes and the lack of difference between the TW set by clinicians evaluating fluid status and the bioimpedance measured normally hydrated weight. Whereas the primary analysis of the trial compared the difference between the TW and the BI-NHW, here we used the distance between them in either direction. Throughout the trial there was close agreement between the TW and the actual weight post-dialysis (see supplemental materials published with the primary outcomes) [6].

To investigate the effects of clustering by centre and subjects on outcomes, a three-level nested model (patient, trial visit and centre) was considered. We then report two intraclass correlations resulting from this multilevel model where the intraclass correlation coefficient expresses the similarity, or relatedness, of observations within a cluster, where a cluster may represent a centre or multiple visits in an individual patient. Using random intercepts for centre and subjects only, the first is the level-3 intraclass correlation at the centre level, which reflects the correlation between differences in the same centre. The second is the level-2 intraclass correlation measured at the subject-within-centre level, which reflects the correlation between differences in the same subject by centre. To investigate whether specific quantifiable practices have a demonstrable association with outcomes, the model was refitted adjusting for age, self-reported sex and co-morbidity score, which was calculated using the externally validated Stoke Comorbidity Index [7–9]. For this post-hoc analysis we did not pre-judge the direction of association in view of the conflicting or lack of evidence.

## Results

### Description of Survey Results

**Fig. 3:**
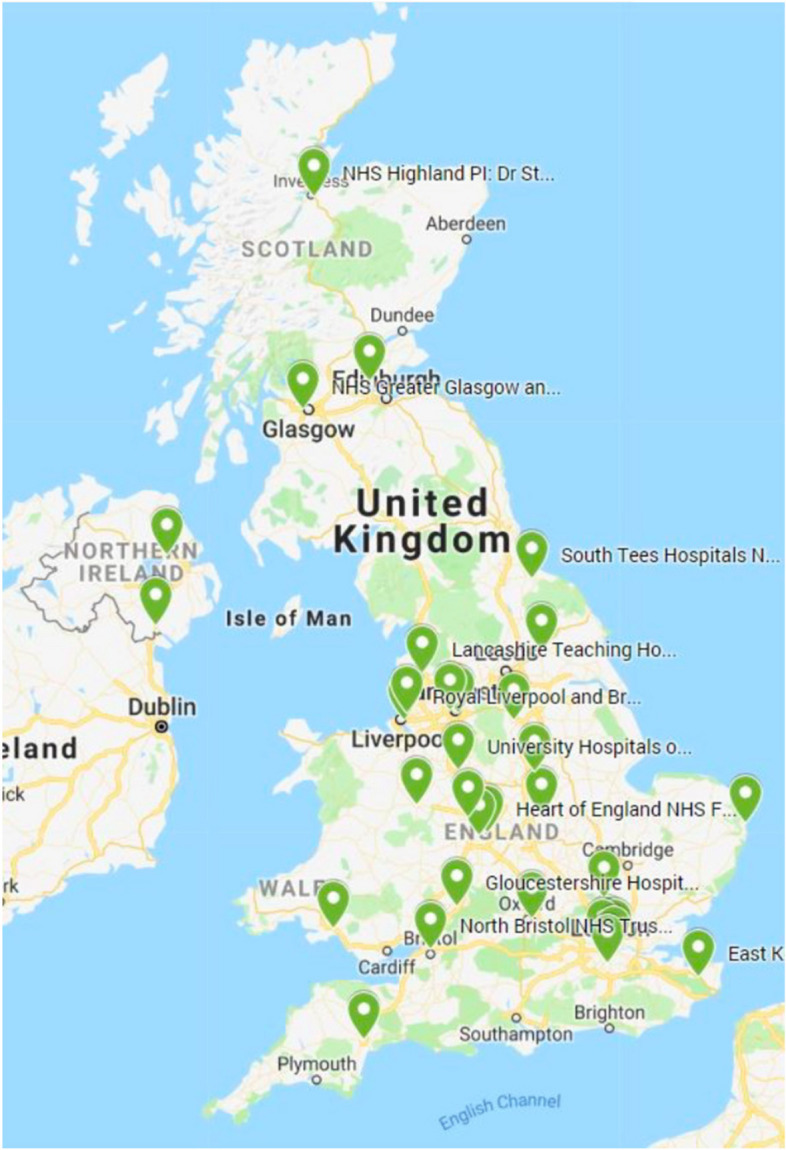
Geographic distribution of centres contributing to the BISTRO trial in the UK

Centres participating in BISTRO were geographically spread throughout the UK (Fig. 3). Of 34 centres selected to participate in the trial, 32 contributed clinical data and 31 of these completed surveys; only 26 completed S1 as five centres joined the trial after 2018. In 10 centres, the survey was completed by the same individual with 80–90% of the responses being the same. The descriptive data for the responses are shown in Table 1. There was little change overall in practice between the two surveys, and where this was observed it could partly be explained by the additional five centres in the second survey. For example the apparent increase in the proportion of centres taking residual kidney function into account in S2 was partly due to this being the practice in three of the five late entry centres. Similarly the apparent difference in the routine use of chest X-rays between the surveys was due to this; only two centres reported using chest X-rays in S2 that did not in S1.

**Table 1:** Descriptive Summary of Survey Results comparing the findings for the first survey (S1) with the second (S2)

Survey Domain	Survey number and date		
**Sodium dialysate concentration**	**S1 (2017–18)**	**S2 (2020–21)**	
The proportion of centres using a standard sodium dialysate concentration	88.5%	67.8%	
The concentration of sodium most frequently used (mmol/L)	138	138	
*(Range)*	*(136–140)*	*(136–140)*	
The proportion of patients (mean/median) that have an individualised dialysate [Na +]	9.3%/1.5	8.9%/0	
*(Range)*	*(0–100%)*	*(0–50%)*	
*(Proportion by category < 10%, > 10% of patients have individualised dialysate [Na*^*+*^*])*	*90%,10%*	*90%,10%*	
*If individualised sodium concentrations are used:*			
Use of a high sodium concentration due to Intradialytic Hypotension (IDH)	35.0%	40.0%	
Use of a high sodium concentration due to hyponatraemia	3.8%	16.7%	
Use of a high sodium concentration due to other reasons	3.8%	0%	
The % of centres that match to plasma sodium	4.8%	19.4%	
The proportion of centres that use sodium profiling/modelling to prevent IHD in patients prone to IDH	39.1%	29.0%	
**Diet and salt intake**			
The proportion of centres with a dedicated dietician	100%	80.6%	
The proportion of centres with an agreed policy on sodium restriction	72.0%	35.5%	
If so, the median advised daily intake of sodium (g) was:	5.2	4.9	
* (Range)*	*(1–24)*	*(2–6)*	
The proportion of centres with an agreed policy on fluid restriction	92.0%	54.8%	
If so, the median advised daily fluid intake (ml) was:	882	850	
* (Range)*	*(500–1500)*	*(500–1500)*	
The proportion of centres with HD nurses trained in fluid management/salt restriction	75%	74.2%	
The proportion of centres who provide written advice to patients regarding intake and restrictions	100%	87.1%	
**Residual Kidney Function**			
The proportion of centres routinely measuring residual kidney function (RKF)	19.2%	29.0%	
The mean interval at which RKF is measured (months)	2.4	3	
*If residual kidney function is measured:*			
The % of centres using RKF to reduce the frequency of dialysis	45.4%	70.0%	
The % of centres using RKF to reduce the length of dialysis sessions	45.4%	70.0%	
**Diuretics and incremental start**			
The proportion of centres who prescribe loop diuretics to the majority of patients	37.5%	36.7%	
The mean typical dose of furosemide per day	131mg	193mg	
* (Range)*	*(40-250mg)*	*(40-500mg)*	
The mean typical dose of bumetanide per day	2.6mg	2.6mg	
* (Range)*	*(2-3mg)*	*(2-6mg)*	
The proportion of centres that commence HD incrementally	36.0%	16.7%	
* Of these*, the proportion that use incremental HD so as to preserve RKF	25.0%	33.3%	
**Fluid assessment**			
The proportion of centres who have a standardised protocol for assessing fluid status in new patients	34.6%	26.7%	
*Whether a protocol is used or not:*			
Centres that routinely use bioimpedence in all patients – not just those in the trial	37.5%	38.4%	
Centres that routinely use chest XRAYs	18.1%	44.0%	
Centres that routinely use Echocardiograms	9.0%	20.8%	
Centres that routinely use Central vein diameter	9.0%	4.3%	
Centres that routinely use Blood volume monitoring	50.0%	53.8%	
Centres that routinely use Lung ultrasound	0%	4.17%	
Centres that routinely use Orthostatic BP to assess dry weight	N/A^a^	46.2%	
The proportion of centres in which assessments are carried out by consultants	100%	90.0%	
The mean proportion of assessments conducted by consultants	49.5%	38.5%	
* (Range)*	*(10–100%)*	*(5–100%)*	
The proportion of centres in which HD dedicated staff grades assess fluid status	50.0%	46.7%	
The mean proportion of assessments carried out by dedicated staff grades	53.7%	38.7%	
* (Range)*	*(0–100%)*	*(10–80%)*	
The proportion of centres in which HD nurses assess fluid status	91.3%	90.0%	
The mean proportion of assessments carried out by HD nurses	43.0%	54.3%	
* (Range)*	*(2–90%)*	*(5–100%)*	
The proportion of centres in which Training grade doctors assess fluid status	60.0%	30.0%	
The mean proportion of assessments carried out by Training grade doctors	20.5%	16.7%	
* (Range)*	*(5–100%)*	*(5–10%)*	
Frequency of reviewing fluid status (median and mode in months)	2	2	
**Fluid management**			
The % of centres in which a consultant prescribes fluid management	92.3%	76.7%	
The mean % of fluid management decisions carried out by consultants	55.0%	47.7%	
* (Range)*	*(10–100%)*	*(5–100%)*	
The % of centres in which a HD dedicated staff grade prescribes	55.0%	26.7%	
The mean % of fluid management decisions carried out by HD dedicated staff grades	52.8%	36.7%	
(*Range)*	*(0–100%)*	*(10–50%)*	
The % of centres in which a HD nurse prescribes	69.6%	70.0%	
The mean % of fluid management decisions carried out by HD nurses	36.8%	54.1%	
* (Range)*	*(20–75%)*	*(10–100%)*	
The % of centres in which a Training grade doctors prescribes	58.9%	30.0%	
The mean % of fluid management decisions carried out by Training grade doctors	11.5%	12.7%	
* (Range)*	*(5–15%)*	*(5–10%)*	
The % of centres with a policy to limit maximum Ultrafiltration (UF) rates	44.0%	30.0%	
If so, what is the mean maximum rate permitted for ultrafiltration (ml/kg/hour* (Range)*	9.1*(1–10)*	14.5*(10–50)*	
And if so, what is the maximum volume removal permitted in a single HD session (Mean and Range)	3527ml *(3000-4800ml)*	3313ml *(3000-4000ml)*	
The % of centres performing isolated ultrafiltration	N/A^a^	90%	
The % of centres with a policy to limit maximum change in target weight	18.2%	16.7%	
**Dialysate temperature**			
The proportion of centres with a standard machine dialysate temperature	96.1%	73.3%	
The most frequent standard machine dialysate temperature (°C )	36	36	
* (Range)*	*(35-37)*	*(35-37)*	
The % of centres who lower dialysate temperature from standard to prevent IDH in patients who are prone to IHD	92.0%	60.0%	
The mean decrease in dialysate temperature compared to standard temperature in patients with IDH (°C)	0.8	0.8	
* Range*	*(0.5-2)*	*(0.5-3)*	

*HD* Haemodialysis, *IDH* Interdialytic hypotension

^a^Not asked in the first survey

There were however inconsistencies in certain domains, most notably the dietary salt and water section, where agreement between the surveys was between 50 and 60% in responses for policies on salt and fluid restriction. For both these measures, S2 reported less restrictive practices and it is of note that this occurred alongside the reported reduction in availability of dedicated dieticians. The exception to this was the strong agreement across the board that patients were given written advice about dietary intake and restrictions.

### Centre-level and patient-level correlations with outcomes

Two thousand, five hundred one fluid assessments from 439 randomised patients with contemporaneous measures of bioimpedance-derived fluid status and pre- and post-dialysis blood pressure were available for analysis and their unadjusted intraclass corrections are summarised in Table 2. Centre-level intraclass correlations for all of the outcomes were extremely low, indicating low probability that centre level practices or policies were having an impact on our outcomes of interest. In contrast, intraclass correlations ranging between 0.12 and 0.47 were observed at the patient level. These were strongest for both pre- and post-dialysis systolic blood pressure.

**Table 2:** Summary of unadjusted centre and patient level intra-class correlation (ICCs)

Outcomes of interest	Centre-Level intra-class Correlation		Patient-Level intra-class Correlation	
	ICC	95%CI	ICC	95%CI
TW-NHW	0.004	0.0001, 0.435	0.273	0.229, 0.321
Pre-dialysis SBP	0.006	0.0002, 0.148	0.472	0.427, 0.517
Pre-dialysis DBP	0.011	0.002, 0.054	0.210	0.174, 0.252
Post-dialysis SBP	0.009	0.001, 0.086	0.388	0.344, 0.434
Post-dialysis DBP	0.013	0.004, 0.044	0.123	0.094–0.159

*SBP* Systolic blood pressure, *DBP* Diastolic blood pressure

### Multi-level analysis of the association between selected fluid management practices and outcomes

Figure 1 shows the distribution of default dialysate sodium concentrations used in the centres. Data completeness was better for S2, but in both surveys a range was observed, with the majority of centres using concentration between 137 and 138 mmol/L. In the multilevel modelling no association was observed with any of the outcomes and dialysate [Na^+^]. The only significant association observed in any of these models was a lower pre-dialysis diastolic blood pressure in older patients with more comorbidity, and a moderate association with male sex (see Table 3). These negative associations, which were present consistently across all the models, were also seen between post-diastolic blood pressure and age and male gender, but not with comorbidity. No associations in any of the models were observed with either pre- or post-dialysis systolic blood pressures.

**Table 3:** Association in Survey 1 between pre-dialysis diastolic blood pressure and default centre dialysate sodium concentration estimated by a multi-level model adjusted for trial visit, age, sex and co-morbidity score

Dialysate [Na^+^] (mmol/L)	Regression coefficient	95% CI	*P* value
135	(Reference)		
136	3.783	–7.028, 14.595	0.493
137	6.932	–3.891, 17.756	0.209
137.5^a^	3.614	–9.482, 16.712	0.589
138	4.204	–6.097, 14.505	0.424
140	6.274	–5.186, 17.734	0.283
Adjusted for:			
Visit (order)	–0.065	–0.175, 0.433	0.237
Age (year)	–0.387	–0.475, –0.299	< 0.001
Sex (M v. F)	–2.400	–0.501, 0.206	0.071
Comorbidity score	–1.501	–2.611, –0.611	0.008

^a^A single site reported this temperature which might have been an error, as in survey 2 they reported 137 mmol/L; its exclusion from this analysis did not affect the result

Figure 2 shows the distribution of dialysate temperatures used, ranging from 35 to 37°C, with better data completeness for S2. No relationship between dialysate temperature and any of the blood pressure measurements was observed. As with the D-[Na^+^] model, a low pre-dialysis diastolic blood pressure was associated with older age, male gender and more comorbidity. In S2, in the analysis of the distance of TW from BI-NHW, when compared to the reference value of 35°C, temperatures of 36° and 36.5° were associated with a significantly more negative coefficient (Table 4), which indicates that in centres using these higher temperatures the patients were closer to their normally hydrated weight. Similar associations were seen for the other temperatures, suggesting that use of a very low dialysate temperature (just one centre in S2) might be associated with patients being further from their BI-NHW. Similar, non-significant trends were observed in the S1 analysis (data not shown). In both the S1 and S2 analyses older patients were more likely to be closer to their BI-NHW, whereas there was a weak association in the opposite direction for those with increased comorbidity.

**Table 4:** Association in Survey 2 between distance of target weight from normally hydrated weight (BI-NHW) and centre default dialysate temperature concentration estimated using a multi-level model adjusted for trial visit, age, sex and co-morbidity score

Temperature Category	Coefficient	95% CI	*P* value
35^o^	(Reference)		
35.5^o^	–0.669	–1.458, 0.119	0.096
36^o^	–0.791	–1.538, –0.044	0.038
36.5^o^	–0.882	–1.640, –0.122	0.023
37^o^	–0.633	–1.561, 0.294	0.181
Adjusted for:			
Visit (order)	–0.003	–0.018, 0.012	0.685
Age (year)	–0.015	–0.025, –0.006	0.001
Gender (M v. F)	–0.052	–0.319, 0.215	0.703
Comorbidity Score	0.059	–0.053, 0.172	0.299

We also tested for other practices associated with fluid assessment, including the use of a standardised protocol for assessing fluid status in new dialysis patients and the routine use of additional methods and techniques (e.g. bioimpedance, chest X-rays, echocardiograms central vein (vena cava) diameter measurement, lung ultrasound and orthostatic hypotension). None of these was consistently associated with the outcome measures of fluid status or blood pressure.

## Discussion

The primary purpose of measuring centre-level clinical practices associated with fluid management in BISTRO was to establish whether a shift in these practices had occurred during the conduct of the trial. Comparing the results of the two surveys revealed that, overall, little change had occurred. There was a suggestion that more attention was being paid to residual kidney function by the end of the trial, which might be interpreted as participation in the trial affecting unit practices, although this was partially explained by the centres joining the trial later and only contributing to the second survey. There was also a suggestion in the second survey that practices were being applied less indiscriminately (e.g. dialysate temperature and sodium concentration, sodium restriction, fluid restriction) although this might, in the case of dietary advice, reflect a reduction in the reported availability of dedicated dieticians. The reason for this cannot be determined from the survey, and may reflect many factors, such a workforce issues related to the COVID pandemic. Also there should be caution in inferring cause an effect here, especially as it is clear that dialysis nurses were empowered to give education on salt and fluid restriction. However the general picture was one of stability of practices during the trial. There was little evidence from either iteration of the survey that centres were using protocols to assess and manage fluid status, despite the fact that the Dialysis Outcomes and Practice Patterns Study has identified having such protocols is associated with better outcomes [4]. However, by virtue of being in the trial all the participants were exposed to the same fluid assessment protocol, a proforma designed to encourage a formalised approach to the complex intervention.

The unadjusted analysis of intraclass correlations for the outcomes of interest demonstrated a clear pattern. The extremely low correlations at the centre level are strongly indicative of a lack of any centre effect, regardless of whether they were linked to the clinical practices we interrogated with the survey or other unmeasured centre effects. This aligned with our primary analysis of the trial, in which fluid assessments were not affected by centre and there was no centre effect on the rate of decline in kidney function [6]. In contrast, we did observe modest correlations at the patient level for each of the outcomes, which were strongest for the systolic blood pressure readings.

Patient-level associations were also seen in the multilevel analyses. There were strong correlations between lower diastolic blood pressure and increasing age and comorbidity using the externally validated Stoke comorbidity score, which is weighted in favour of cardiovascular disease, in particular ischemic heart disease, peripheral vascular disease (including cerebrovascular disease) and heart failure, all indicative of the arterial wall stiffness that is highly prevalent in dialysis patients. Taken together, these observations further reinforce the strong relationship between cardiovascular damage and outcomes and the implication is that any current centre-level fluid management practices have little or no effect on this.

This conclusion is further reinforced by the lack of association with any of the practices that we examined more specifically in our adjusted analyses. The lack of any observable effect of a default dialysate sodium concentration in either of the surveys is suggestive but not conclusive. We do not know for certain whether the unit-level policy on dialysate sodium concentration was universally applied to trial participants as this was not recorded routinely in the trial and it is likely that we had sufficient power to test this. The ongoing cluster randomised RESOLVE trial (NCT02823821), which is testing two sodium concentrations in a large multi-national collaboration, will hopefully clarify this important question. Equally, the possible association we observed between a low reported dialysate temperature and a greater deviation from the normally hydrated weight should be treated with caution. This was only seen in survey 2, in which only one centre reported using a default temperature of 35°C, so this could be a type 1 error. The recent MyTEMP trial did not show an survival benefit of reducing the dialysate temperature [10, 11], and if anything the current analysis suggests the centre-wide application of very low temperatures is not helpful.

Our analysis has a number of limitations. We cannot rule out errors in survey completion, although the reproducibility, especially when surveys were completed by the same individual, was good. In this post hoc analysis linking specific practices to outcomes, there is likely to be lack of statistical power and we cannot be certain that the centre-level default practices were necessarily universally applied to all trial participants. We did not analyse the impact of anti-hypertensive medications in the survey as this was adjusted for in the primary trial analysis and only collected at baseline. It would have been complex to analyse and when designing the surveys we decided to restrict it to direct effects on fluid status. Nor did we ask about the use of hemodiafiltration, although this data ws collected on an individual basis at the start of the trial (and not different by trial allocation). Some of the practices, such as who was doing fluid assessments, were not possible to analyse due to their complexity and substantial overlap in responses. It may also be argued that routine pre- and post-dialysis readings are not the optimal method for assessing blood pressure in haemodialysis patients, with preferred methods being interdialytic ambulatory blood pressure monitoring [2, 12]. Bioimpedance derived normally hydrated weight is only one dimension of fluid status. In our primary analysis, where we used the net difference between TW and BI-NHW, increasing age was associated with more overhydration, so the reduced absolute difference in either direction observed here implies that there is less variation with age even if, on average, age is associated with overhydration. We did not have sufficient power to undertake sub-group analyses. Perhaps most importantly, these patients by participating in a trial that used a standardised approach to assessing fluid status meant that any other centre-level practices effects were over-ridden. Finally, it should be remembered that patients in BISTRO were new to dialysis and by definition had residual kidney function at the start of treatment. It may be that effects of centre-level practices are attenuated in this selected group.

In summary, interpretation of the results of the BISTRO trial is not affected by centre-level effects or practices related to fluid management. The lack of a centre effect may reflect the use of a standardised approach to fluid assessment in this group of patients with residual kidney function. There is significant variation in practices related to fluid management in the UK and further adequately powered cluster-randomised trials are needed to establish their importance for clinical management. There is scope to further develop, and evaluate the use of protocols to manage fluid status in haemodialysis patients.

## Supplementary Information

Supplementary Material 1.

## Data Availability

The trial data are available to investigators under the conditions of a data sharing agreement. This will include group- and individual-level fully anonymized data. Applications should be made to the corresponding author.
