# Supplementary material for: Centre-level fluid management practices in the BISTRO trial and their lack of association with participant fluid status and blood pressure in non-anuric haemodialysis patients
Source: BMC Nephrol. 2024 Nov 6;25:398. doi: 10.1186/s12882-024-03837-y (PMC11542461; doi:10.1186/s12882-024-03837-y)
Supplement: Supplementary file 1 — Supplementary Material 1. [file 12882_2024_3837_MOESM1_ESM.pdf]

59700

BISTRO Trial
v2.0, 19-Feb-2020

TeleForm ID

Centre ID: ☐☐☐☐☐

Page completed by (Name):
PLEASE PRINT

Signature:

Date ☐☐ / ☐☐ / ☐☐☐☐ **(DD/MM/YYYY)**

Trial Stage:

☐ Beginning of trial ☐ Closed Recruitment
☐ Closed Follow Up ☐ End of Trial - Date ☐☐/☐☐/☐☐☐☐

SAFE STAPLE AREA IS BETWEEN THE TWO MARKED ARROWS

Section 1: Sodium Dialysate Concentration

a. Is there a standard sodium dialysate concentration in your unit? Yes ☐ No ☐

b. What is the concentration of sodium used most frequently? ☐☐☐.☐ mmol/L

c. What proportion of patients have an individualised sodium concentration? ☐☐☐%

d. If individualised sodium concentrations are used:

d1a. If low, what reason? ....

d1b. If high, which of the following reasons? **(cross as many as applicable)**

☐ Intradialytic Hypotension
☐ Hyponatraemia
☐ Other ....

d2. Is your practice to match the plasma sodium? Yes ☐ No ☐

e. For patients prone to intradialytic hypotension (IDH), do you use sodium profiling/modelling to prevent IDH?
Yes ☐ No ☐

Section 2: Nutrition and Sodium Intake

a. Does your HD unit have a dedicated dietitian? Yes ☐ No ☐

a1. If yes, how much time per patient do they have each month? ☐☐:☐☐ (Hours:Minutes)

b. Do you have a policy on sodium restriction? Yes ☐ No ☐

b1. If yes, what is the advised daily intake? ☐☐ grams/day

c. Do you have a policy on fluid restriction? Yes ☐ No ☐

c1. If yes, what is the advised daily intake? ☐☐☐☐ ml/day

d. Are all your dialysis nurses on the HD unit trained in fluid management/salt restriction? Yes ☐ No ☐

e. Are patients given written advice about dietary intake and restrictions? Yes ☐ No ☐

Section 3: Measurement of Residual Kidney Function

a. Do you routinely measure residual kidney function on your unit? Yes ☐ No ☐

a1. If Yes, how frequently **(cross one box only)**

☐ Monthly
☐ Every 2 months
☐ Every 3 months
☐ Every 6 months
☐ Only when clinically indicated

a2. If Yes, do you use residual kidney function to reduce the:

a2.1 Frequency of the dialysis? Yes ☐ No ☐

a2.2 Length of dialysis sessions? Yes ☐ No ☐

SAFE STAPLE AREA IS BETWEEN THE TWO MARKED ARROWS

For office use only			
Received at CTU Initials	Logged Initials	Data entry Initials	Quality checked Initials

Unit Level Practice Survey
Fluid Management

TeleForm ID

Centre ID: ☐☐☐☐☐

Section 4: Diuretics

a. Are the majority of patients with residual kidney function routinely prescribed loop diuretics? Yes ☐ No ☐

b. What is the typical dose of: 1. Furosemide ☐☐☐ mg

2. Bumetanide ☐ mg

c. Do you currently use any other diuretics **(cross as many as applicable)**

☐ Metolazone ☐ Aldosterone inhibitors

☐ Thiazides ☐ Other...

Section 5: Incremental Dialysis

a. Is it routine practice in your unit to commence HD incrementally? Yes ☐ No ☐

a1. If Yes, is this to preserve residual kidney function? Yes ☐ No ☐

b. In the context of incremental start what percentage of patients in your unit do:

1. 1 session per week? ☐☐☐ %
2. 2 sessions per week? ☐☐☐ %

Section 6: Assessment and Prescription

a. Do you have a standardised protocol for assessing fluid status in new patients? Yes ☐ No ☐

b. Protocol or not, in addition to clinical assessment do you <u>routinely</u> use:

a. Bioimpedance	Yes ☐	No ☐
b. Chest, X-Ray	Yes ☐	No ☐
c. Echocardiogram	Yes ☐	No ☐
d. Central vein diameter	Yes ☐	No ☐
e. Blood volume monitoring	Yes ☐	No ☐
f. Lung ultrasound	Yes ☐	No ☐
g. Orthostatic BP measurement to assess dry weight	Yes ☐	No ☐

c. Who assesses fluid status on your unit?

a. Consultants	Yes ☐	No ☐	If Yes, what percentage of assessments	☐☐☐ %
b. HD dedicated staff grades	Yes ☐	No ☐	If Yes, what percentage of assessments	☐☐☐ %
c. HD nurses	Yes ☐	No ☐	If Yes, what percentage of assessments	☐☐☐ %
d. Training grade doctors	Yes ☐	No ☐	If Yes, what percentage of assessments	☐☐☐ %

d. How often is fluid status reviewed? **(cross one box only)**

☐ Not at all ☐ Monthly ☐ 2 Monthly

☐ 3 Monthly ☐ 6 Monthly ☐ Only when clinically indicated

For office use only			
Received at CTU Initials	Logged Initials	Data entry Initials	Quality checked Initials

Section 7: Fluid Management Strategies

a. Who prescribes fluid management on your unit? **(cross as many as applicable)**

a. Consultants	Yes ☐	No ☐	If Yes, what percentage of assessments ☐☐☐ %
b. HD dedicated staff grades	Yes ☐	No ☐	If Yes, what percentage of assessments ☐☐☐ %
c. HD nurses	Yes ☐	No ☐	If Yes, what percentage of assessments ☐☐☐ %
d. Training grade doctors	Yes ☐	No ☐	If Yes, what percentage of assessments ☐☐☐ %

b. Do you have a policy to limit the maximum UF rates in your unit? Yes ☐ No ☐

c. If Yes:

c1. What is the maximum rate permitted? ☐☐ ml/kg/hour

c2. What is the maximum volume permitted in a single HD session? ☐☐☐☐ ml

c3. Do you perform isolated ultrafiltration? Yes ☐ No ☐

d. If you are changing the target weight, do you have a policy on the maximum change you would make at a time?

Yes ☐ No ☐

d1. If Yes, what is the maximum change you would make at a time **(choose either Kg or % for your answer)**

☐☐.☐ kg **OR** ☐☐☐ %

Section 8: Dialysate Temperature

a. Do you have a standard machine dialysate temperature in your centre?

Yes ☐ No ☐

b. What is your standard dialysate temperature?

☐☐.☐ °C

c. For those prone to IDH, do you lower dialysate temperature from standard to prevent IDH?

Yes ☐ No ☐

d. If yes, how much below standard?

☐☐.☐ °C

End of Survey

Thank you for taking the time to fill in this survey, your answers will be very useful to us.

Please check that you have answered all of the questions and return the survey in the sealed envelope provided.

Please **DO NOT** damage (ie, staple through) any of the black square corner stones of this form.

For office use only			
Received at CTU Initials	Logged Initials	Data entry Initials	Quality checked Initials
